# The Diagnostic and Prognostic Roles of Combined Expression of Novel Biomarkers in Lung Adenocarcinoma and Lung Squamous Cell Carcinoma: An Immunohistochemical Study

**DOI:** 10.30699/IJP.2020.130944.2452

**Published:** 2020-12-21

**Authors:** Mohamed Ali Alabiad, Ola A. Harb, Mohamed Abozaid, Ahmed Embaby, Doaa Mandour, Rehab Hemeda, Amany Mohamed Shalaby

**Affiliations:** 1 *Pathology Department, Faculty of Medicine, Zagazig University, Zagazig, Egypt*; 2 *Chest Department, Faculty of Medicine, Zagazig University, Zagazig, Egypt*; 3 *Internal Medicine Department, Faculty of Medicine, Zagazig University, Zagazig, Egypt*; 4 *Clinical Oncology and Nuclear Medicine Department, Faculty of Medicine, Zagazig University, Zagazig, Egypt*; 5 *Histology and Cell Biology Department, Faculty of Medicine, Tanta University, Tanta, Egyp* *t*

**Keywords:** Diagnosis, Immunohistochemistry, Lung adenocarcinoma, Lung squamous cell carcinoma

## Abstract

**Background & Objective:**

Diagnosis and discrimination of lung adenocarcinoma (LUAD) from lung squamous cell carcinoma (LUSC) is critical to select the appropriate treatment regimen as recently targeted therapies require accurate subtyping of nonsmall-cell lung carcinoma (NSCLCs). There are currently several biomarkers that could be used for differentiation between LUAD and LUSC, but they have less sensitivity, specificity, and clinical applicability. The aim of this study was to assess the diagnostic and prognostic values of CLCA2, SPATS2, ST6GALNAC1, and Adipophilin tissue expression in the tissues retrieved from LUAD and LUSC patients using immunohistochemistry.

**Methods:**

The current study was performed on the samples retrieved from sixty primary lung masses that were diagnosed as LUAD and LUSC. Immunohistochemistry was performed by using a panel of CLCA2, SPATS2, and ST6GALNAC1. We assessed the diagnostic roles of the studied markers in the discrimination between LUAD and LUSC and their prognostic values.

**Results:**

SPATS2 and CLCA2 were expressed higher in LUSC than LUAD. ST6GALNAC1 and Adipophilin showed higher expression in LUAD than LUSC (*P***<**0.001). The sensitivity and specificity of CLCA2, SPATS2, ST6GALNAC1 and Adipophilin in adequate subtyping and reaching the accurate diagnosis was 100%. We found only significant difference in survival rate between the patients with negative and positive CLCA2 expression (*P*=0.038 and *P*=0.019, respectively).

**Conclusion:**

The combination of biomarkers of CLCA2, SPATS2, ST6GALNAC1, and Adipophilin may lead to an appropriate subtyping of lung cancer and reaching accurate diagnosis with the highest sensitivity and specificity.

## Introduction

Non-small cell lung cancer (NSCLC) accounts for nearly 89% of all lung cancers. They are further categorized as lung adenocarcinoma (LUAD) and lung squamous cell carcinoma (LUSC) which are the two major pathological subtypes of lung cancer, forming about 45% and 25% of cases, respectively (1). The precise histopathological diagnosis and discrimination of LUAD from LUSC is critical to select the appropriate treatment regimen as recently targeted therapies require accurate sub-typing of NSCLCs (2). There are many differences between LUAD and LUSC in their molecular profiling and histological characteristics, but small biopsies with a limited number of tumor cells and tumors with uncertain structures caused by poor differentiation or necrosis make difficult a precise diagnosis relying on the routine histopathological evaluation. Thus, immunohistochemistry is now widely recommended in the clinical practice (1). There are currently several biomarkers that were assessed by immunohistochemistry and were found to be useful in the differentiation of LUAD from LUSC, but they have less sensitivity, specificity, and clinical applicability (2). The chloride channel accessory 2 (CLCA2) is a protein which belongs to the family of chloride-sensitive proteins (3). Its basic functions include chloride conductivity regulation, and it can play a role in epidermal differentiation and skin malignancies (4). The spermatogenesis associated serine-rich 2 (SPATS2) has been reported to play a critical role in the spermatogenesis and development of testicular germ cells and its paralogue, SPATS2L was reported to have a role in genome-wide association response genes (2). The ST6 (alpha-N-acetyl-neuraminyl-2, 3-beta-galactosyl-1, 3)-N-acetylgalacto saminide alpha-2,6-sialyltransferase 1 (ST6GALNAC1) is a member of the sialyltransferase family reported as being overexpressed in several cancers and is correlated with the cancer metastases (2). Adipophilin is a transporter of small lipid droplets in non-adipogenic cells. The role of Adipophylin in cancer has recently been studied and it has been found to play both diagnostic and prognostic roles in many cancers (5). 

The aim of this study was to assess the diagnostic and prognostic values of CLCA2, SPATS2, ST6GALNAC1, and Adipophilin tissue expression in the tissues retrieved from LUSC and LUAD patients using immunohistochemistry.

## Materials and Methods

This randomized retrospective study was done on the samples retrieved from sixty primary lung masses patients. We retrospectively collected paraffin blocks and patients’ data from the Pathology Department, Faculty of Medicine, Zagazig Universityfrom February 2015 to February 2020. All patients’ data such as age, sex, progression and survival, and tumor histopathological findings such as size, number, and the site of the lung masses were recorded in addition to recording all significant clinical and radiological findings.

The biopsy from variable sizes of lung masses was performed by bronchoscopic biopsy or by transthoracic core biopsy. The hematoxylin and eosin (H&E)-stained slides of tumor tissue were reviewed for confirmation of the diagnosis according to the most recent classification of the World Health Organization (WHO) (6), and for the selection of the best sites for the staining of biomarkers using immunohistochemistry.

**Fig. 1:**
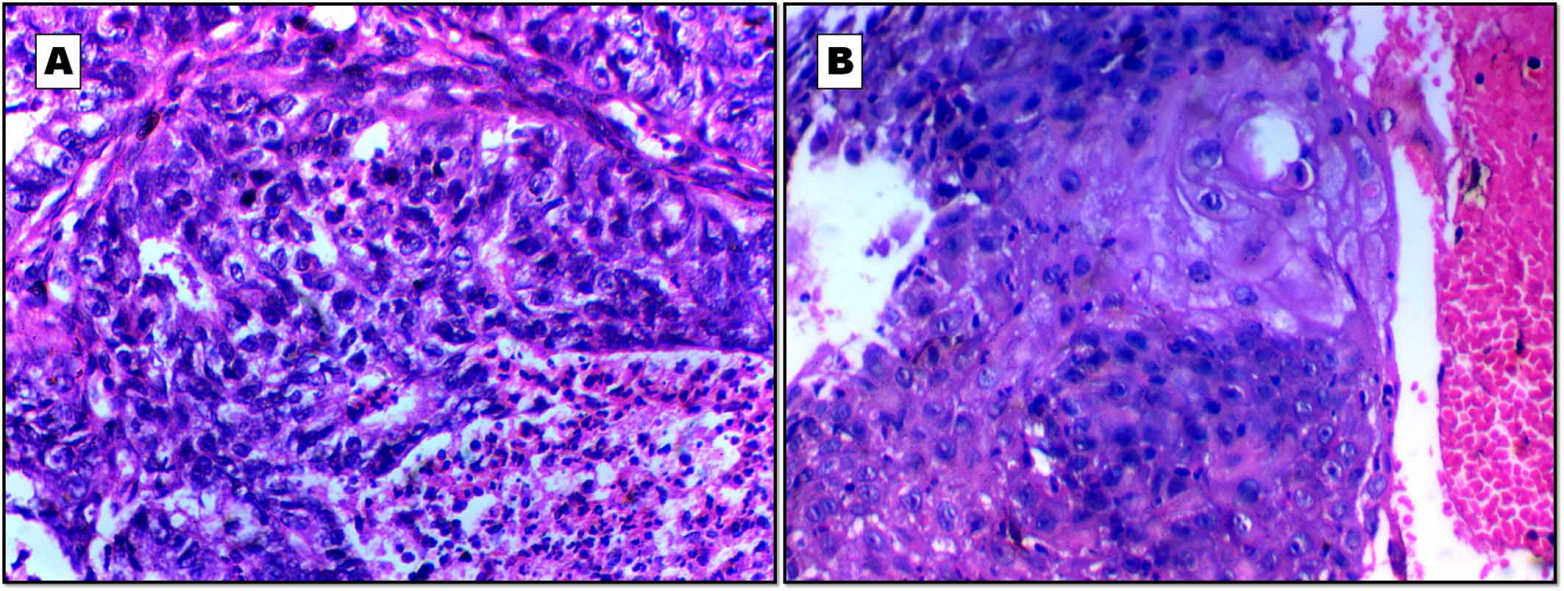
Histophathological description of the studied lung cancer samples. A: Poorly differentiated lung adenocarcinoma (LUAD) with focal areas of glandular formation and focal necrosis. B: Moderately differentiated lung squamous cell carcinoma (LUSC) with intra and extracellular keratinization

Diagnosis of LUAD and its differentiation from LUSC, particularly in poorly differentiated foci, was done by detection of any remaining areas of differentiation into any histopathological subtype. LUAD was generally characterized by the presence of areas of glandular formations, and intracellular mucin. LUSC differentiation was characterized by the presence of intracellular and/or extracellular keratin in addition to the presence of intercellular bridges (Figure 1). The inclusion criteria were a) all primary NSCLC samples and b) all true cut (small) biopsies, and the exclusion criteria were a) ll NSCLC with less material, b) all NSCLC with necrotic areas, and c) all small biopsies with insufficient biopsy material.

**Immunohistochemistry (IHC)**

Immunohistochemistry was performed by using a panel of Rabbit Polyclonal antibodies against CLCA2 (LS-C664626) (LifeSpan BioSciences), SPATS2 (ab122495) (Abcam), ST6GALNAC1 (MBS7053229) (MyBioSource's Products) and Adipophilin (393A-1) (Sigma-Aldrich). Briefly, 3-4 μm thick paraffin-embedded tissues was cut, deparaffinized, and dehydrated in graded alcohol at 100%-50%. Then the targeted antigen retrieval was performed followed by the application of the primary antibodies. Two histopathologists examined the stained slides. With the help of a combined scoring system (sum of staining intensity and percentage of positive cells quantification), the semi-quantitative immunoreactivity analysis for the IHC markers was conducted in the neoplastic cells. The intensity of the staining was scored as 0 for absent, 1 for weak, 2 for moderate, and 3 for strong. The positive cells were quantified as percentage of the total number of neoplastic cells and were calculated as less than 5% = 0, 5% - 25% = 1, 26% - 50% = 2, 51% - 75% = 3, greater than 75% = 4. For each case, an immunoreactivity score was generated as a percentage of positive tumor cells and staining intensity, generating a score ranging from 0 to 12. A case was considered positive if the score was equal to or greater than 2; otherwise, the case was considered negative (2), (3), (5). The classification of each case into its subtype was based on the immunohistochemical profile. We calculated the sensitivity, specificity, and accuracy of each stained biomarker and the combined values of all the studied biomarkers by comparing between the immunostaining results of the included samples.

**Statistical Analysis**

The collected data were statistically analyzed using the Statistical Package for the Social Sciences (SPSS 24 Inc., Chicago, IL, USA). Shapiro Walk test was used for testing data for normal distribution. Qualitative data were represented as frequencies and relative percentages. We used Chi-square (χ2) and Fisher exact tests to calculate the differences between qualitative variables. We expressed quantitative and non-parametric data as median and range. 

**Survival Analysis**

We estimated progression-free and overall survival rates using the Kaplan and Meier method and compared them using the log-rank test. The overall survival rate (OS) was calculated as the time from disease diagnosis to the time of death or to time of last follow up or time of ending the study. We estimated progression-free survival rate (PFS) as the time from initiation of treatment date to the date of starting disease progression or to the time of last follow up or time of ending the study. The P-value≤0.05 and P-value<0.001 indicate significant and highly significant differences, respectively, while P-value>0.05 indicates a non-significant difference. 

## Results

The samples from 60 cases of poorly differentiated lung cancer were included, which were divided into 30 cases of LUSC and 30 cases of LUAD. Demographic, clinicopathological, and follow-up parameters of all included patients were detailed in Table 1. 

**Table 1:** Clinico-pathologic and follow-up characteristics of the studied lung cancer patients (N=60)

Characteristics				Histopathological subtype			
		Total N=60		Squamous N=30		Adenocarcinoma N=30	
		N	%	N	%	N	%
Age group	<65 years	27	45.00%	4	13.30%	23	76.70%
	≥65 years	33	55.00%	26	86.70%	7	23.30%
Sex	Male	39	65.00%	30	100.00%	9	30.00%
	Female	21	35.00%	0	0.00%	21	70.00%
Comorbidities	No	31	51.70%	12	40.00%	19	63.30%
	Yes	29	48.30%	18	60.00%	11	36.70%
Smoking	No	25	41.70%	0	0.00%	25	83.30%
	Yes	35	58.30%	30	100.00%	5	16.70%
Grade	1	12	20.00%	5	16.70%	7	23.30%
	2	37	61.70%	20	66.70%	17	56.70%
	3	11	18.30%	5	16.70%	6	20.00%
Size	5-7cm	19	31.70%	2	6.70%	17	56.70%
	>7cm	41	68.30%	28	93.30%	13	43.30%
Site	Upper lobe	17	28.30%	6	20.00%	11	36.70%
	Middle lobe	24	40.00%	16	53.30%	8	26.70%
	Lower Lobe	14	23.30%	6	20.00%	8	26.70%
	All lung	5	8.30%	2	6.70%	3	10.00%
Malignant pleural or pericardial effusions	No	48	80.00%	22	73.30%	26	86.70%
	Yes	12	20.00%	8	26.70%	4	13.30%
Stage	Stage IIB	17	28.30%	10	33.30%	7	23.30%
	Stage IIIA	14	23.30%	1	3.30%	13	43.30%
	Stage IIIB	7	11.70%	4	13.30%	3	10.00%
	Stage IV	22	36.70%	15	50.00%	7	23.30%
LN metastasis	Negative	16	26.70%	11	36.70%	5	16.70%
	Positive	44	73.30%	19	63.30%	25	83.30%
Distant metastases	No	38	63.30%	15	50.00%	23	76.70%
	Yes	22	36.70%	15	50.00%	7	23.30%
T	T2b	14	23.30%	0	0.00%	14	46.70%
	T3	27	45.00%	19	63.30%	8	26.70%
	T4	19	31.70%	11	36.70%	8	26.70%
N	0	16	26.70%	11	36.70%	5	16.70%
	1	10	16.70%	0	0.00%	10	33.30%
	2	14	23.30%	3	10.00%	11	36.70%
	3	20	33.30%	16	53.30%	4	13.30%
M	M0	38	63.30%	15	50.00%	23	76.70%
	M1a	7	11.70%	5	16.70%	2	6.70%
	M1b	15	25.00%	10	33.30%	5	16.70%
Response to treatment	PD	10	16.70%	7	23.30%	3	10.00%
	SD	15	25.00%	11	36.70%	4	13.30%
	PR	35	58.30%	12	40.00%	23	76.70%
Response to treatment	NR	15	25.00%	10	33.30%	5	16.70%
	OAR	45	75.00%	20	66.70%	25	83.30%
Progression	No	39	65.00%	16	53.30%	23	76.70%
	Yes	21	35.00%	14	46.70%	7	23.30%
Death	Alive	31	51.70%	14	46.70%	17	56.70%
	Dead	29	48.30%	16	53.30%	13	43.30%

**Cytoplasmic Expression of Both CALCA2 and SPATS2**

The expression of CALCA2 and SPATS2 was found higher in LUSC than LUAD. Cytoplasmic and membranous ST6GALNAC1 expression and cytoplasmic Adipophilin expression were higher in LUAD compared to LUSC (*P***<**0.001). 

**Fig. 2:**
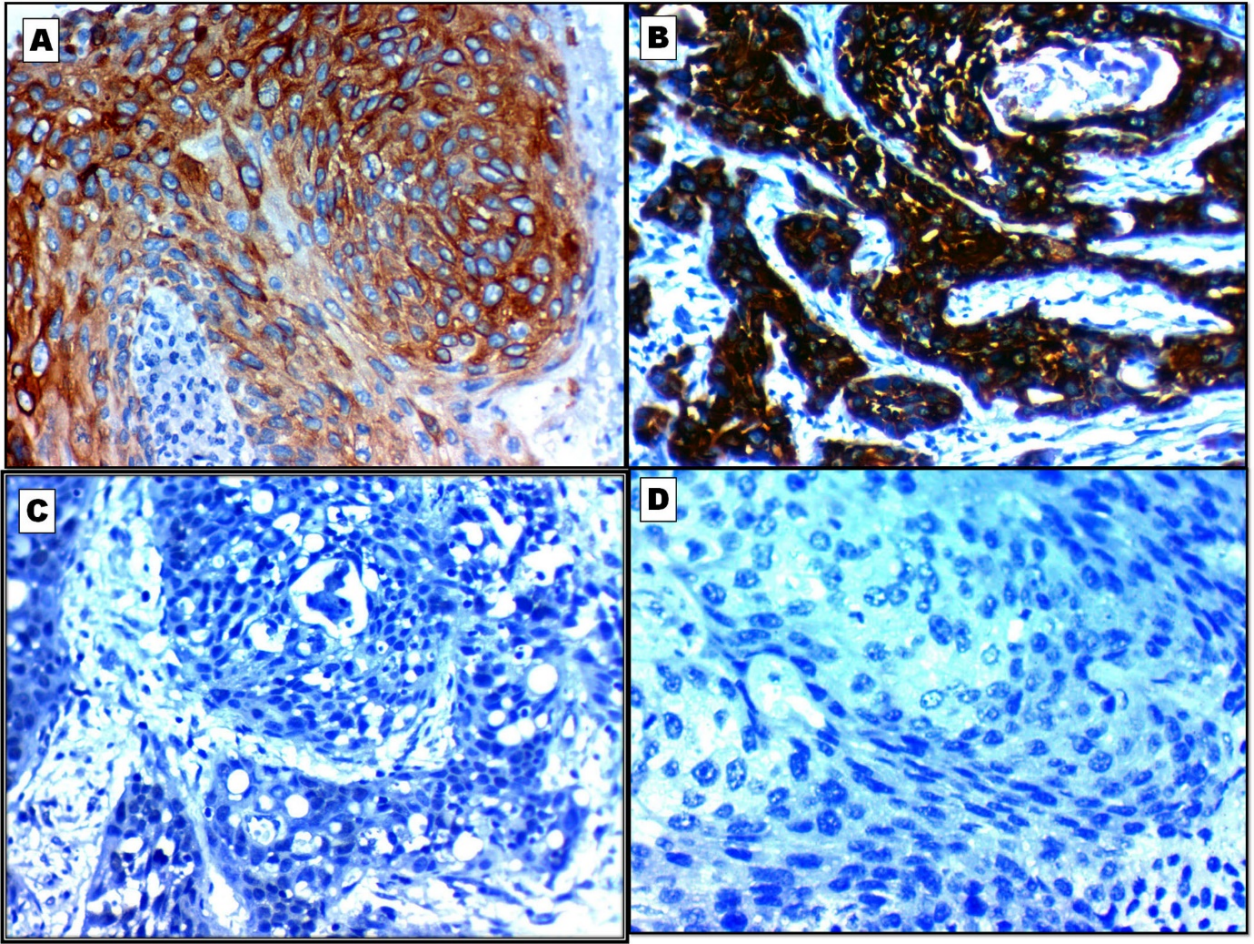
Immunohistochemical profile of lung squamous cell carcinoma (LUSC) according to the results of the studied markers. A: Diffuse cytoplasmic expression of CLCA2 in LUSC X400. B: Diffuse cytoplasmic expression of SPATS2 in LUSC X400. C: Negative expression of ST6GALNAC1 in LUSCX400. D: Negative expression of Adipophilin in LUSCX400

**Fig. 3:**
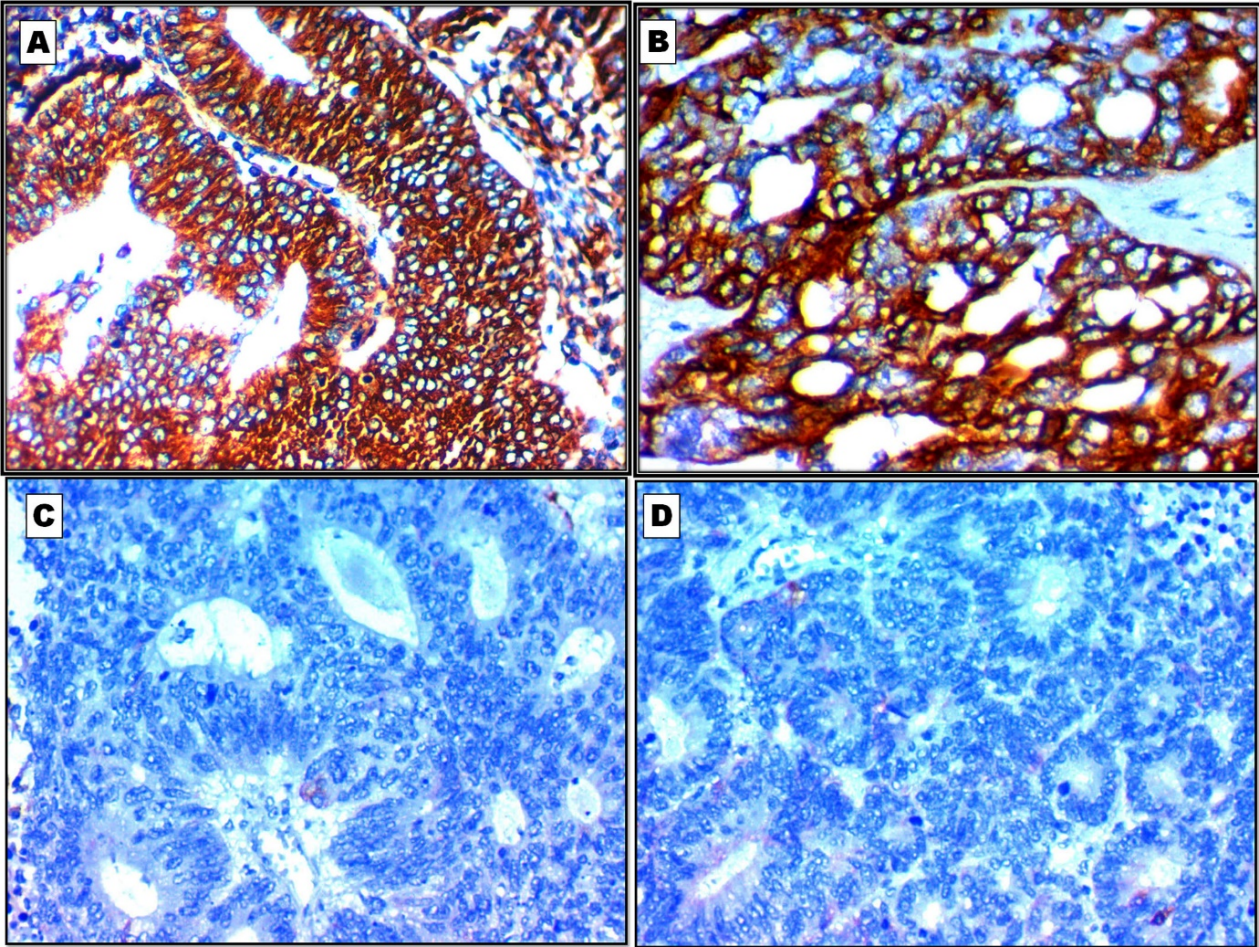
Immunohistochemical profile of lung adenocarcinoma (LUAD) according to the results of studied markers. A: Diffuse cytoplasmic and membranous expression of ST6GALNAC1 in LUAD X400. B: Diffuse cytoplasmic expression of Adipophilin in LUAD X400. C: Negative expression of CLCA2 in LUADX400. D: Negative expression of SPATS2 in LUAD X400

The diagnostic performance of CLCA2, SPATS2, ST6GALNAC1, and Adipophilin expression in the differentiation between LUAD and LUSC are shown in details in Table 2 and Figures 2 and 3.

**Table 2:** Diagnostic performance of CLCA2, SPATS2, ST6 GALNAC1 and Adipophilin expressions in differentiation between Lung adenocarcinoma (LUAD) and lung squamous cell carcinoma (LUSC)

Marker expression		Histopathological subtype				Total N=60		*P*	Sensitivity (95% CI)	Specificity (95% CI)	AUC (95% CI)	PPV (95% CI)	NPV (95% CI)
		Squamous		Adenocarcinoma									
		N=30		N=30									
		N	%	N	%	N	%						
CLCA2*	Negative	5	16.70%	29	96.70%	34	56.70%	**<0.001**					
	Positive	25	83.30%	1	3.30%	26	43.30%		(65- 94%) 80%	(82- 99%) 90%	(0.795 - 0.962)	(78.333% - 99.425%)	(72.211% - 92.829%)
SPATS2*	Negative	3	10.00%	29	96.70%	32	53.30%	**<0.001**					
	Positive	27	90.00%	1	3.30%	28	46.70%		(73- 97%) 85%	(82- 99%) 94%	(0.838 - 0.982)	(79.659% - 99.466%)	(76.729% - 96.592%)
Adipophilin^£^	Negative	27	90.00%	3	10.00%	30	50.00%	**<0.001**					
	Positive	3	10.00%	27	90.00%	30	50.00%		(62- 96%) 78%	(73- 97%) 86%	(0.751 - 0.952)	(65.597% - 94.395%)	(75.899% - 96.258%)
ST6GALNAC1^£^	Negative	28	93.30%	1	3.30%	29	48.30%	**<0.001**					
	Positive	2	6.70%	29	96.70%	31	51.70%		(82- 99%) 84%	(77- 99 %) 89%	(0.861 - 0.990)	(79.143% - 98.227%)	(80.262% - 99.484%)
CLCA2, SPATS2, ST6 GALNAC1, and Adipophilin									100%	100%	100%	100%	100%

**Target Genes Tissue Protein Expression in LUSC and LUAD**

The sensitivity and specificity of CLCA2 expression for LUSC diagnosis and its differentiation from LUAD were 80% and 90%, respectively (Table 3) and the sensitivity and specificity of SPATS2 expression for LUSC diagnosis and its differentiation from LUAD were 85% and 94%, respectively (Table 3). The sensitivity and specificity of ST6GALNAC1 expression for the diagnosis of LUAD and its differentiation from LUSC were 84% and 89%, respectively (Table 3). The sensitivity and specificity of Adipophilin expression for the diagnosis of LUAD and its differentiation from LUSC were 98% and 86%, respectively (Table 3).

The sensitivity and specificity of CLCA2, SPATS2, ST6GALNAC1, and Adipophilin in adequate subtyping of poorly differentiating lung cancer and reaching accurate diagnosis was 100%.

**Associations Between CLCA2, SPATS2, ST6GALNAC1, and Adipophilin Expression and Survival of Included NSCLC Patients**

**Fig. 4:**
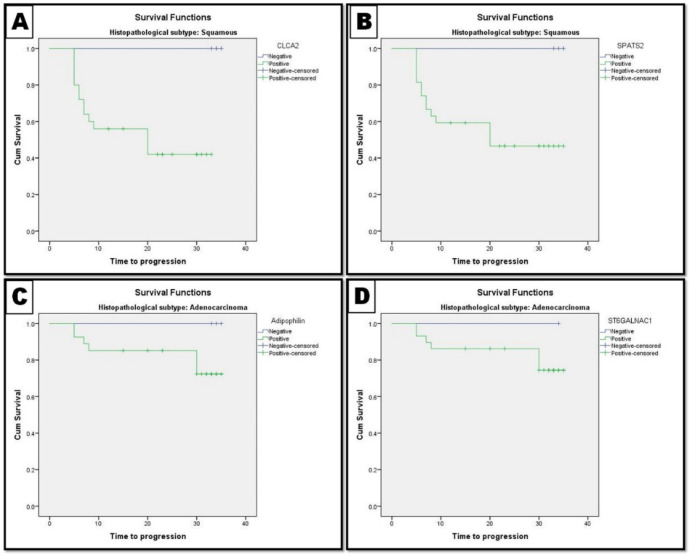
Progression free survival rate (PFS) of the patients according to the studied biomarkers. A and B: PFS rate of LUSC patients and association with CLCA2, SPATS2 expression. C and D: PFS rate of LUAD patients and association with ST6GALNAC1 and Adipophilin expression

**Fig. 5:**
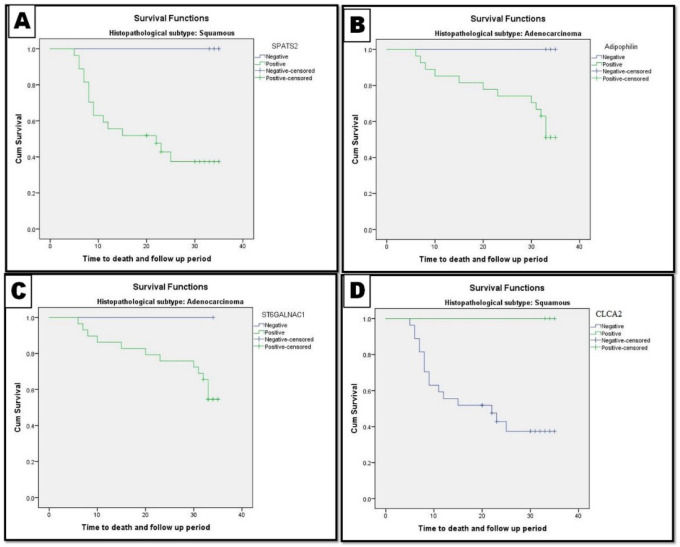
Overall survival rate (OS) of the patients according to the studied biomarkers. A and B: OS rate of LUSC patients and association with CLCA2, SPATS2 expression. C and D: OS rate of LUAD patients and association with ST6GALNAC1 and Adipophilin expression

Significant differences were found in the PFS and OS rates between patients with negative and positive CLCA2 expression (*P*=0.038 and *P*=0.019, respectively). However, no statistically significant difference was observed in PFS and OS rates between patients with negative and positive SPATS2, ST6GALNAC1, and Adipophilin expression. Table 3 and Figures 4 and 5.

**Table 3:** Progression-Free survival and overall survival rates of the included patients and association with expression of the studied markers

Marker	Histopathological subtype		Progression-Free Survival Analysis					Overall Survival Analysis				
			N of Events	Censored		PFS Rate%	*P*	N of Events	Censored		OS Rate%	*P*
				N	Percent				N	Percent		
CLCA2	Squamous (N=30)	Negative (N=5)	0	5	100.0%	100%	**0.038**	0	5	100.0%	100%	**0.019**
		Positive (N=25)	14	11	44.0%	42.0%		16	9	36.0%	31.5%	
	Adenocarcinoma (N=30)	Negative (N=29)	7	22	75.9%	74.5%	**0.586**	13	16	55.2%	54.6%	**0.439**
		Positive (N=1)	0	1	100.0%	100%		0	1	100.0%	100%	
SPATS2	Squamous (N=30)	Negative (N=3)	0	3	100.0%	100%	**0.128**	0	3	100.0%	100%	**0.09**
		Positive (N=27)	14	13	48.1%	46.6%		16	11	40.7%	37.4%	
	Adenocarcinoma (N=30)	Negative (N=29)	7	22	75.9%	74.5%	**0.586**	13	16	55.2%	54.6%	**0.439**
		Positive (N=1)	0	1	100.0%	100%		0	1	100.0%	100%	
Adipophilin	Squamous (N=30)	Negative (N=27)	14	13	48.1%	46.6%	**0.128**	16	11	40.7%	37.4%	**0.09**
		Positive (N=3)	0	3	100.0%	100%		0	3	100.0%	100%	
	Adenocarcinoma (N=30)	Negative (N=3)	0	3	100.0%	100%	**0.325**	0	3	100.0%	100%	**0.16**
		Positive (N=27)	7	20	74.1%	72.4%		13	14	51.9%	51.2%	
ST6GALNAC1	Squamous (N=30)	Negative (N=28)	14	14	50.0%	48.6%	**0.226**	16	12	42.9%	40.0%	**0.18**
		Positive (N=2)	0	2	100.0%	100%		0	2	100.0%	100%	
	Adenocarcinoma (N=30)	Negative (N=1)	0	1	100.0%	100%	**0.586**	0	1	100.0%	100%	**0.439**
		Positive (N=29)	7	22	75.9%	74.5%		13	16	55.2%	54.6%	

## Discussion

Being the most commonly identified disease and the leading cause of tumor mortality, lung cancer has been projected to be responsible for more than one million reported diagnoses and almost a million deaths all over the world (7). LUAD and LUSC are the two main clinical subtypes of lung cancer that make up the overwhelming majority of lung cancers identified, although there are also discrepancies in their competent biochemical mechanisms and characteristics, as well as treatment approaches (8).

Over the recent years, the usage of selective treatment based on the molecular and histological features of cancer types has become a common procedure. In the form of non-small cell lung cancer, it is important to differentiate between LUAD and LUSC to pick the most appropriate medication regimen (9) 

However, this is often a problem for the pathologists in tumors with undefined structures leading to poor differentiation, necrosis, tiny biopsies, or cytology with a restricted number of tumor cells. It is impossible to render an accurate diagnosis based on the H&E staining alone. At this point, the mixture of immunohistochemical findings may be re-diagnosed; hence, immunohistochemical staining is still advised and commonly utilized in the clinical procedures (10).

Currently, a range of effective immune-histochemical markers have been introduced to differentiate LUAD from LUSC, including TTF-1, napsin-A, p63, and CK5/6. Nevertheless, owing to the absence of a systematic study of various subtypes of lung cancer, there might also be un-discovered markers of higher sensitivity, specificity, and application utility (11-16).

There are three CLCA proteins (CLCA1, CLCA2, and CLCA4), CLCA2 is a chloride conductance protein, belongs to the calcium-sensitive family, and considered as one of the p53 targets that negatively controls the development, proliferation, and invasion of malignant cells. Moreover, CLCA2 expression is correlated with markers modifications of the extracellular matrix in lung SCC (17-19). 

Our immunohistochemical research showed that the CLCA2 protein expression level was the substantially higher frequency in the LUSC relative to that in LUAD (80% sensitivity and 90% specificity). These findings indicate that CLCA2 can be a novel useful immunohistochemical marker for the differential diagnostics between LUSC and LUAD. In line with our findings, Shinmura *et al. *(3) reported that CLCA2 protein expression in differentiation between LUSC and LUAD showed 64.1% sensitivity and 99.1% specificity. 

The expression of CLCA2 has been verified to correlate with poor clinical outcomes in the breast cancer patients, while the impact of reduced CLCA2 expression on survival has not yet been studied in the patients with other types of cancer. Our current study showed that impairment of CLCA2 protein expression status was a poor prognostic factor in the patients with LUSC; also, CLCA2 protein expression was reported by Shinmura *et al. *(3) to be a poor prognostic factor in the female patients with LUSC.

The SPATS2 gene was first identified to encode a polypeptide comprising 545 amino acid residues in the testis involved in sperm growth and production. Subsequently, the researchers observed that SPATS2 had also been expressed in 25 human tissues. Recent research has shown that SPATS2 is strongly expressed in squamous cell carcinoma but rarely in non-lepidic AD (20-21).

Our study showed that SPATS2 protein production was higher in LUSC than in LUAD, where the sensitivity of measurement of SPATS2 expression for the diagnosis of SCC was 85%, and the specificity was 94%. Such results suggest that SPATS2 can be a new, effective immunohistochemical marker for the differential diagnosis between LUSC and LUAD. Takamochi* et al.| *(2) and Osmani* et al. *(22) who studied on discrimination between LUSC and LUAD, found that the sensitivity and specificity of SPATS2 in detecting lung SCC were (63% and 100%) and (67% and 100%), respectively. This lower sensitivity may be due to few cases and restrictions on the included cases where they include a specific subgroup of poorly differentiated squamous cell carcinoma (PDSCC). 

ST6GalNAC1 is one of the candidate enzymes for the Sialyl-Tn synthase, which is strongly expressed in many human carcinomas and is consistent with carcinoma aggressiveness and poor prognosis (23-25).

Our analysis found that ST6GalNAC1 protein expression was substantially higher in LUAD than in LUSC (84% sensitivity and 89% specificity). Such results suggest that ST6GalNAC1 could be a novel marker for the differential diagnosis between LUAD and LUSC. In accordance with our results, Takamochi* et al. *(2) observed the sensitivity and specificity of ST6GalNAC1 in LUAD separation from LUSC at 67% and 100%, respectively; such minor modifications could be attributed to a small range of cases and limitations on the eligible cases where they have a particular PDSCC subgroup.

Our survival review of the patients with evaluated SPATS2 and ST6GALNAC1 protein expression found no statistically meaningful gap in the progression-free survival or overall survival levels between the patients with negative and positive SPATS2 and ST6 GALNAC1 expression.

Metabolic changes, as well as mutations, are defining characteristics in cancer biology. Cancer cells may change their metabolic pathways to maximize the energy needed for replication or expansion, initially influencing glucose metabolism but increasingly in lipid cholesterol. High lipid content in cancer cells is also an indication of aggressive behavior. Up-regulation of the lipid synthesis pathway has been documented in several malignancies, including breast cancer, retinoblastoma, lung cancer, and colon cancer (26-29).

Visualization of small lipid droplets (LDs) is better encouraged by the immunohistochemical production of Adipophilin, a vector for tiny LDs in non-Adipogenic cells. The function of Adipophilin in cancer has recently been explored and it is not only a diagnostic marker but also an important prognosis predictor for other cancers, though research on the prognosis importance of Adipophilin in lung cancer has been minimal (30-32).

Our study showed that the expression of Adipophilin was higher in LUAD than in LUSC (84% responsiveness and 89% specificity). Such results indicate that Adipophilin could be a detective novel marker for the differential diagnosis of LUAD and LUSC. Following our progress, Shin* et al. *(5) and Zhang* et al. *(33) indicated that the degree of Adipophilin expression was significantly higher in the lung adenocarcinoma than in squamous cell carcinoma.

## Conclusion

In the current study, we used four novel biomarkers and evaluated their expression using immunohistochemistry in the tissue sections from poorly differentiated lung cancer with different histopathological subtypes aiming at adequate diagnosis and subtyping them to LUSC and LUAD which are considered as the most common subtypes that have different treatment modalities, particularly in small samples and necrotic samples with few viable cells. We have concluded that the combined usage of CLCA2, SPATS2, ST6GALNAC1, and Adipophilin may serve as potential markers for improving the diagnostic utility for distinguishing between LUSC and LUAD reaching a sensitivity and specificity up to 100%.

In the current study, we used novel biomarkers that have not been already reported as a panel in the differentiation between LUSC and LUAD and assessed their expression using immunohistochemistry. The combination of these four markers improves the sensitivity and specificity of detection up to 100% that can be valuable in poorly differentiated carcinoma and small biopsies. This is the strength point of our study. 

 Additionally, we have assessed the prognostic values of those markers and correlated their expression with patients’ survival, which might help predict the prognosis of lung cancer and discover novel targeted therapies for such lethal cancer.

Limitations and weakness points of our work are the relatively few numbers of cases, single-center evaluation of the patients and their samples in addition to using only immunohistochemistry for evaluating the tissue protein expression. Performing a large-scale study involving a large number of patients’ populations using different methods of marker evaluation such as genetic studies are recommended to confirm our results.
